# Metagenomic analysis reveals the microbiome and antibiotic resistance genes in indigenous Chinese yellow-feathered chickens

**DOI:** 10.3389/fmicb.2022.930289

**Published:** 2022-09-07

**Authors:** Yibin Xu, Yulin Huang, Lijin Guo, Siyu Zhang, Ruiquan Wu, Xiang Fang, Haiping Xu, Qinghua Nie

**Affiliations:** ^1^Lingnan Guangdong Laboratory of Modern Agriculture, State Key Laboratory for Conservation and Utilization of Subtropical Agro-Bioresources, College of Animal Science, South China Agricultural University, Guangzhou, Guangdong, China; ^2^Guangdong Provincial Key Lab of Agro-Animal Genomics and Molecular Breeding and Key Lab of Chicken Genetics, Breeding and Reproduction, Ministry of Agriculture, Guangzhou, Guangdong, China

**Keywords:** yellow-feathered broiler, metagenomics, microbiome, antibiotic resistance gene, binning

## Abstract

Yellow-feathered chickens have great nutritional value and are widely and traditionally used in China, on an industrial scale as broilers. The presence of intestinal microbes has been shown to correlate with poultry performance and serves as an essential reservoir of antibiotic resistance genes (ARGs). Antibiotic resistance is a major public health concern. Here, we investigated functional characteristics of the gut microbiome of indigenous Chinese yellow-feathered chickens (the Huiyang Bearded, Xinghua, Huaixiang, Zhongshan Shanlan, Qingyuan Partridge, and Yangshan chickens) through metagenomic sequencing and reconstructed 409 draft genomes, including 60 novel species and 6 novel genera. Furthermore, we assessed the functions of the intestinal microbial communities and examined the ARGs within them. The results showed that the microbial populations of yellow-feathered broilers were primarily dominated by Bacteroidetes and Firmicutes at the phylum level and *Bacteroides* at the genus level. Furthermore, the Qingyuan Partridge chicken showed a significantly higher abundance of *Prevotella* than the other five breeds of chicken. Principal coordinates analysis indicated significant differences in the structures of microbial communities and ARGs, based on the binary Jaccard distance, among the six chicken breeds. Moreover, 989 ARGs conferring tetracycline, multidrug, and aminoglycoside resistance were identified, which represented more than 80% of the faecal resistomes; the most abundant gene in the yellow-feathered chickens was *tet(Q)*. In addition, we found the greatest abundance of resistance genes in Xinghua chickens, indicating that Xinghua chickens are highly resistant to antibiotics. Overall, our findings revealed differences in the gut microbial community structure of indigenous Chinese yellow-feathered broiler breeds and the composition and characteristics of ARGs and antibiotic resistance that enabled us to reconstruct the yellow-feathered chicken gut microbial community genomes. The current data significantly improves our knowledge of the gut microbiome and antibiotic resistance of popular broiler breeds in China.

## Introduction

In the past several decades, the broiler industry has developed rapidly in China and, after the United States, China is the second-largest producer of broilers. In the broiler industry, yellow-feathered and white-feathered chickens are mainly bred. Yellow-feathered chickens account for around 50% the chicken meat produced in China, with an annual production exceeding 4 billion birds (Li et al., 2019). Yellow-feathered broiler production is concentrated in the southern area of China, led by Guangdong Province. Yellow-feathered chickens are also known as three-yellow chickens because of their characteristic yellow beaks, feathers, and feet (Du et al., 2016). Their importance is evidenced by the incredible increase in demand. For instance, the production of yellow-feathered chicken meat in China reached 4,445 kt in 2015, representing 31.8% of the national broiler meat yield (Zheng et al., 2016). Among the various indigenous chicken breeds in Guangdong, six have been preserved because of their distinctive appearance and varied genetic diversity; these are include the Huiyang Bearded, Xinghua, Huaixiang, Zhongshan Shalan, Qingyuan Partridge, and Yangshan chickens (China National Commission of Animal Genetic Resources, 2011). These six varieties might have different genetic backgrounds.

The gut microbial genome is considered to be the host’s second genome, which is closely related to the growth and development of the host (Zhu et al., 2010a). The intestinal microbiome plays an important role in host metabolism, immunity, and physiology (Kohl, 2012; Diaz Carrasco et al., 2019). Metagenomic sequencing has become a powerful method for analysing microbial communities. Compared to 16S rRNA gene sequencing, metagenomics can produce assemblies of higher resolution. Based on metagenomics, less abundant taxa and their functional potential can be identified, which could reveal more microbial diversity within and between samples. The intestinal microbiota is considered a crucial organ that plays an integral role in maintaining host health by modulating several physiological functions, including nutrition, metabolism, and immunity (Stanley et al., 2013; Mancabelli et al., 2016). The digestive process is strongly linked to the gut microbiota. Consequently, nutrient absorption, feed digestibility, energy harvesting, and productivity are influenced by microbiota composition and diversity (Oakley et al., 2014; Clavijo and Flórez, 2018). Currently, the intestinal microbiomes, as vast reservoirs of antibiotic resistance genes (ARGs), have been the focus of several investigations, and the composition and characteristics of ARGs have identified. The gut microbial genomes of the six above-mentioned breeds remain largely unknown and require further characterisation. With the rapid decline in production costs and the intensive use of antibiotics, an increasing number of sub-therapeutic doses of antibiotics are being used in the breeding industry to promote growth and prevent diseases, which places high pressure on Chinese poultry production. The global consumption of antibiotics used for chickens, pigs, and cattle is estimated to increase by 67%, from 63,151 t in 2010 to 105,596 t in 2030 (Van Boeckel et al., 2015). Improper use of antibiotics in various environments has led to the spread of ARGs in China (Qiao et al., 2018; Glendinning et al., 2020; Feng et al., 2021). The frequent use of antibiotics in livestock animals and humans results in the propagation of ARGs and is becoming a major global health issue.

In this study, we analysed the caecum microbiota, using whole metagenome sequencing, to accomplish the following main goals: (1) investigate the taxonomic abundance among different breeds of broilers through assembly and alignment; (2) functionally annotate the microbial genomes through the Kyoto Encyclopedia of Genes and Genomes (KEGG) and evolutionary genealogy of genes—Non-supervised Orthologous Groups (eggNOG), and Carbohydrate-Active enZymes (CAZy) databases and determine functional abundance; (3) identify antibiotic resistance genes in the caecum microbiota; and (4) reconstruct draft genomes of intestinal bacteria of yellow-feathered chickens. These data improve our understanding of the chicken caecum microbiome and could be a valuable resource for studying antibiotic resistance in indigenous Chinese chickens.

## Materials and methods

### Sample collection

We collected caecal content samples from 24 representative broilers of six indigenous breeds from commercial chicken farms in China, including Huiyang Bearded (HZHXC), Xinghua (FKXHC), Huaixiang (XYHXC), Zhongshan Shanlan (ZSSLC), Qingyuan Partridge (QYMC), Yangshan (YSC) (Supplementary Table 1). All experimental chickens were healthy and received no probiotic or antibiotic therapy before sample collection. The broilers were slaughtered by cervical dislocation and the abdomen was opened. The caecum was excised, and its contents were collected and preserved in sterile containers. These samples were stored at –80°C until DNA extraction. Animal experiments were performed in accordance with the protocols approved by the Institutional Animal Care and Use Committee of the South China Agricultural University (approval number SCAU#2020C029). Animal procedures followed the regulations and guidelines established by this committee, and efforts were made to minimise animal suffering.

### DNA extraction, library construction, and sequencing

DNA from each sample was extracted using the PowerSoil^®^ DNA Isolation Kit following the manufacturer’s instructions. DNA concentration and purity were measured using a Nanodrop 2000 spectrophotometer and agarose gel electrophoresis, respectively. The Bioruptor Pico Sonication System (ultrasonication) was used to fragment the qualified DNA samples, and amplicon sequencing was used for library construction. The constructed library was used to examine library quality, and the Illumina sequencing platform was used for metagenomic sequencing of the qualifying library. The experiments were conducted at Biomarker Technologies Co., Ltd. (Beijing, China).

### Gene prediction and taxonomy annotation

The Trimmomatic (version 0.33, pleading:3 trailing:3 sliding window:50:20 minlen:120) and Bowtie2 (version 2.2.4, –seed 123456 -I 200 -X 1000 –un-conc) software were used to perform quality control and host filtering on the raw reads obtained from sequencing (Bolger et al., 2014). After clean reads were obtained, the MEGAHIT software (version 1.1.2) (Li et al., 2015) was used to filter contig sequences shorter than 300 bp for metagenomic assembly, and gene prediction was performed using MetaGeneMark (version 3.26)^1^ (Zhu et al., 2010b). Information on the gene set for each sample was obtained using CD-HIT (version 4.6.6) by maintaining a clustering threshold of 95% and coverage threshold of 90%. For taxonomy annotation, metagenomic reads from each sample were compared against the non-redundant protein database of National Centre for Biotechnology Information, using DIAMOND (*E*-value ≤ 1e-5).

### Functional annotation

Kyoto Encyclopedia of Genes and Genomes, eggNOG, and CAZy are the most frequently used databases for the studying functional properties of microbial population (Kumar et al., 2020). The KEGG databases include important pathway information about the biological system and provide a way to understand the microbial functions in hosts from metagenomic datasets. The eggNOG database is regarded as a significant public resource for functional annotation and is based on orthologous groups of proteins at different taxonomic levels. Here, we mapped the amino acid sequences of the gene catalog into the proteins in the eggNOG (version 4.0) (Powell et al., 2014) and KEGG (03/2017) (Kanehisa et al., 2017) databases, using the DIAMOND software with default parameters, and then selected the highest-scoring annotated hit. In addition, we used the HMMER software (version 3.0, if alignment >80aa, use *e*-value < 1e-5, otherwise use *e*-value < 1e-3, covered fraction of HMM >0.3) to compare the protein sequences of non-redundant genes with the CAZy database (version 6.0) to identify carbohydrate-active enzymes in the genome. The non-redundant gene set of the gut microbiome in the yellow-feather broilers was further searched against the Comprehensive Antibiotic Resistance Database (Alcock et al., 2020) to assess the presence of antibiotic resistance genes using the RGI software (version 4.2.2, perfect, strict) with an 80% identity cutoff (Alcock et al., 2020).

### Metagenomic binning

To reconstruct microbial genomes from metagenomic data, co-assembly was further performed by dividing 24 samples into 6 groups using MEGAHIT (version 1.1.2, –continue –kmin-2 –k-list 21,29,39,59,79,99,119,141 –min-contig-len 200). The standard for the grouping of metagenomes were based on various breeds. We used three computational methods MetaBat (version 2.12.1) (Kang et al., 2019), MaxBin (version 2.2.6) (Wu et al., 2014), and CONOCOCT (version 1.0.0) (Alneberg et al., 2014) for all contigs. The three sets of bins were further refined using DAS_Tool (version 1.1.2, –search_engine diamond –write_bins 1 –score_threshold 0) (Sieber et al., 2018) to produce a combined and robust bin set. The resulting bins sets from all samples were then pooled and de-replicated using the dRep software (version 3.0.0, -sa 0.95 -comp 80 -nc 10, -comp) (Olm et al., 2017) to generate genomes. Finally, the genome quality was estimated using CheckM (version 1.0.12) (Parks et al., 2015) to calculate genome integrity and contamination. All genomes with completeness of ≥80% and contamination of ≤10% were retained for the subsequent analyses. All bins were dereplicated at 95% average nucleotide identity (ANI) using dRep (v3.0.0). CompareM (version 1.0.1.2)^2^ was used to calculate the average amino acid identity (AAI) among the bins. If the ANI output by GTDB-Tk was < 95%, or if an ANI was not output by GTDB-Tk, the genome was determined to be a novel specie. Genera were defined as novel if all bins clustered at 60% AAI were not assigned a genus by GTDB-Tk. For taxonomic lineages of the reconstructed bins, the GTDB-Tk (version 1.2.0) (Chaumeil et al., 2019) software was used to infer taxonomic annotation for bins based on the Genome Taxonomy Database (GTDB, Release 95). All phylogenetic trees were constructed using the ggtree package in Python (version 3.6.1) and visualised using iTOL (version 6.1.1) (Letunic and Bork, 2021).

### Statistical analysis

Statistical comparisons were performed using one-way analysis of variation tests, and data were obtained using Prism (version 8.0; GraphPad Software, San Diego, CA, United States). The relative abundance of ARG was defined using the unit of “ppm,” namely, one hit of the ARG per million reads (Yang et al., 2013). Statistical significance was set at *P* < 0.05. The α- and β-diversities of the genus, KO, COG, and CAZy profiles were calculated for each sample and visualised data analysis was performed using BMKCloud.^3^

## Results

### Metagenomic sequencing and gene prediction

**FIGURE 1:**
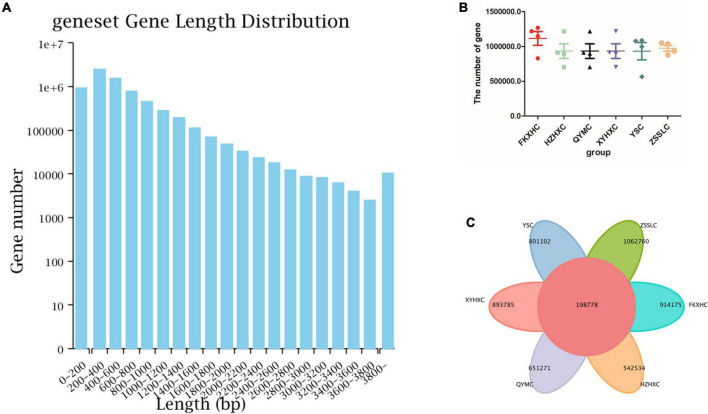
Gene abundance statistics. **(A)** Gene length distribution, *X*-axis: length of the genes, *Y*-axis: number of genes of the total genes. **(B)** Scatter dot plot showing the difference in gene numbers among the six groups. **(C)** Venn diagram showing the numbers of common and unique genes among the six groups. Data are expressed as means ± SEM.

Metagenomic sequencing generated 291 billion raw reads, corresponding to 123 Gbp of raw data from the caecum contents of the 24 broilers. Low-quality read filtering was performed for each sample dataset to obtain clean reads (Supplementary Table 2). After assembling and predicting the sequencing data, information about the reads, such as the length of the open reading frame (ORF) and N50 of each broiler, was obtained as shown in Table 1; the gene length distribution of the non-redundant gene set is shown in the form of a histogram (Figure 1A). We identified 7.11 million non-redundant genes, with an average ORF length of 551 bp. Moreover, we investigated the difference in genes number among the six groups and found that the number of non-redundant genes was higher in FKXHC than in the other five groups; however, this difference was not significant, as shown in the scatter plot (Figure 1B). The number of common and unique genes among the six groups is shown in Figure 1C, where 198,778 genes were found to be common in both regions.

**TABLE 1:** Sequenced reads analysis and assembly statistics.

Sample	Contig Num.	Total Len. (bp)	N50^1^ (bp)	GC (%)	Mapped^2^ (%)
FKXHC1	392222	555133757	1777	48.05	94.65
FKXHC2	433139	628311260	1884	49.23	93.86
FKXHC3	262011	458872158	2874	51.35	95.88
FKXHC4	407587	608707582	2009	48.44	94.35
HZHXC1	237593	361955020	2072	48.56	88.32
HZHXC2	296745	484357564	2374	48.34	95.15
HZHXC3	308913	464894041	2005	48.71	93.88
HZHXC4	295257	476343903	2362	51.55	94.65
QYMC1	219833	401060089	3327	48.47	94.28
QYMC2	239798	355326828	1943	48.01	88.95
QYMC3	321474	525704855	2419	48.74	94.64
QYMC4	263335	426235271	2359	50.54	93.82
XYHXC1	394324	549891671	1734	47.23	92.43
XYHXC2	379211	540337865	1832	48.05	94.05
XYHXC3	401311	571101588	1812	50.01	92.6
XYHXC4	385463	546185646	1823	48.97	92.38
YSC1	237314	226163115	893	46.98	77.19
YSC2	375498	508359646	1646	49.05	91.58
YSC3	338497	498029370	1936	47.65	93.23
YSC4	370584	528209061	1811	47.8	91.82
ZSSLC1	282670	442677074	2215	48.64	94.43
ZSSLC2	348071	512786581	1914	48.06	95.04
ZSSLC3	315508	464274920	1903	48.85	94.97
ZSSLC4	348665	518056690	1978	47.58	95.03

^1^N50 is the shortest contig length that needs to be included for covering 50% of the genome.

^2^Mapped: the comparison rate of sequencing reads and assembly contigs.

### Microbial taxonomy annotation

**FIGURE 2:**
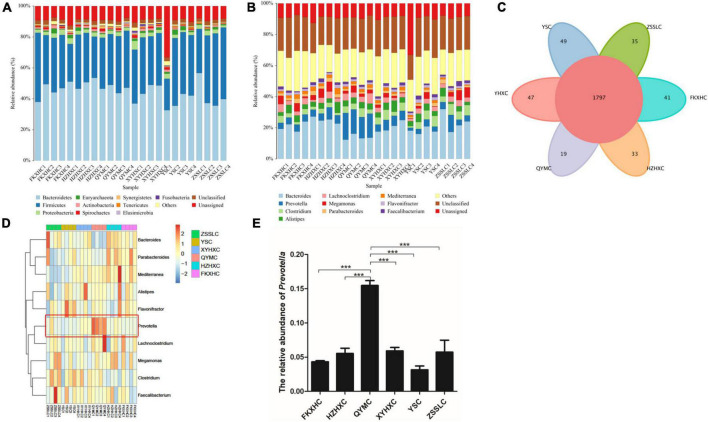
Taxonomic annotation. **(A)** Relative abundance at phylum level. **(B)** Relative abundance at genus level. **(C)** Venn diagram showing the number of common and unique genus among the six groups. **(D)** Heatmap representation of taxonomy abundance of the Top 10 dominant genera. **(E)** Histogram showing the difference in the relative abundance of *Prevotella* among the six groups. Data are expressed as means ± SEM, ***p < 0.001.

**FIGURE 3:**
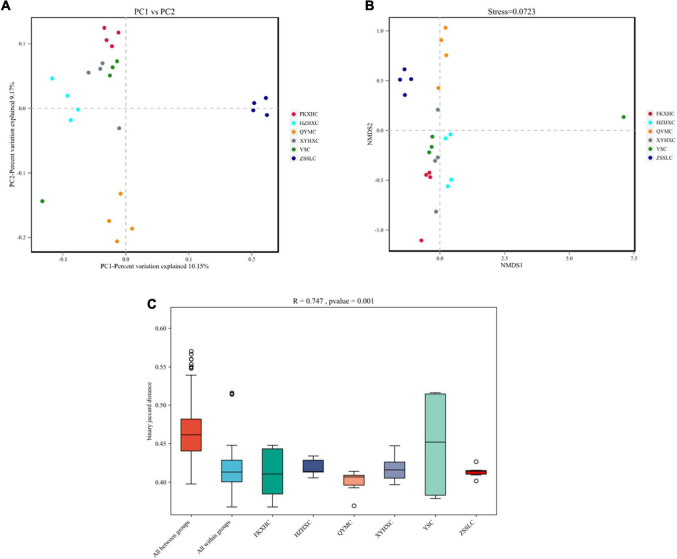
Beta-diversity analysis. **(A)** PCoA plot and **(B)** NMDS plot of binary-jaccard distances among six groups. **(C)** ANOSIM plots for variations among six groups.

The assembled gene sets were aligned against the non-redundant protein database to obtain the microbial population composition and relative abundance information. The distributions of individual samples at the level of phylum and genus are shown in Figures 2A,B. At the phylum level, Bacteroidetes and Firmicutes were the dominant phyla in all samples, accounting for 75–98% of the total bacterial community, with Bacteroidetes commonly dominant. At the genus level, 3,072 genera were identified across all groups (Supplementary Table 3), and 1,797 genera were common to all groups (Figure 2C). *Bacteroides* and *Prevotella* were the dominant genera in the QYMC group (13.9 and 15.5%, respectively), and the dominant genus in the other five groups was *Bacteroides*. Interestingly, we compared the abundance of the top 30 genera in the six groups and found that only the QYMC group had a significantly higher level of *Prevotella* than the other five groups (Figures 2D,E). To distinguish between the variations in host gut microbiomes, the α- and β-diversities of the microbial communities among different chicken groups were also evaluated. The Chao1 index (abundance within samples) demonstrated that the number of genera in the XYHXC group was higher than that in the other groups; however, the difference was not statistically significant. The Shannon index (diversity within samples) of the caecum microflora was more diverse in the HZHXC and ZSSLC groups than the rest (Supplementary Figure 1). In addition, the results of principal coordination analysis (PCoA) and non-metric multidimensional scaling (NMDS) for dimension reduction analysis, based on the binary Jaccard distance, revealed a dissimilarity among bacterial communities of all groups (Figures 3A,B). The closer the sample points are on the scatter chart, the greater the similarity of the composition. The analysis of similarities is a non-parametric test that assesses whether variation between groups is significantly greater than variation within groups, which helps to evaluate the reasonability of the division of groups (*R* = 0.747, *P* = 0.001) (Figure 3C).

### Functional annotation

**FIGURE 4:**
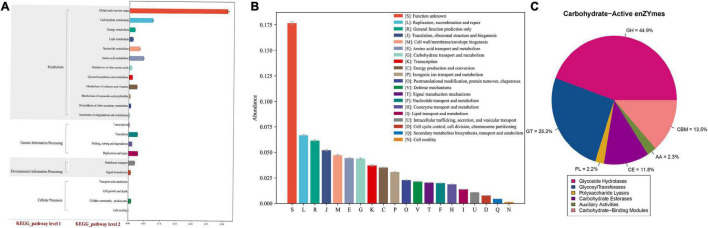
Functional annotation. **(A)** Statistical map of KEGG metabolic pathway-related functional genes at the level 2, *X*-axis: the relative content of the corresponding functional gene number, *Y*-axis: the classification content of KEGG level 1 and level 2. **(B)** Histogram of eggNOG functional gene function classification, *X*-axis: the relative content of the corresponding functional gene number, *Y*-axis: the classification content of eggNOG. **(C)** Pie chart of the proportion of various carbohydrate enzyme.

We then predicted the functional and metabolic pathways of the non-redundant genes in the gut microbiomes of broilers that were sequenced based on the KEGG, eeggNOG, and CAZy databases. The KEGG pathway analysis revealed that a large number of pathways belonged to metabolism groups (77.0%), including carbohydrate (10.3%), amino acid (8.6%), nucleotide (6.2%), metabolism of cofactors and vitamins (6.1%), energy (4.4%), glycan biosynthesis and metabolism (2.7%), and lipid (2.4%) (Figure 4A). The eggNOG analysis showed that most of the gene functions remained unclear (18%), while the known functions were relatively more abundant in replication, recombination, and repair (6.7%); translation, ribosomal structure, biogenesis (5.3%); and cell wall (4.8%); and amino acid transport and metabolism (4.6%) (Figure 4B). Lastly, annotation using the CAZy database revealed that most enzymes were classified as glycoside hydrolases (GHs, 44.9%), followed by glycosyltransferases (GTs, 22.5%), polysaccharide lyases (PLs, 13.5%), and carbohydrate esterases (CEs, 11.8%) (Figure 4C).

Furthermore, we performed comparative analyses of α-diversity in individuals at the KEGG orthology (KO) level, cluster of orthologous groups (COG) level, and family level of CAZy (Supplementary Figure 2). There was no significant difference between the Chao1 and Shannon indices at the KO and COG levels, except that the Chao1 index at the COG level was significantly different between the XYHXC and QYMC groups (Supplementary Figure 2). Moreover, the Shannon index showed that diversity was similar in all groups at the KO and COG levels, and the XYHXC group had significantly higher diversity than other groups at the family level of CAZy.

### Antibiotic resistance gene annotation

**FIGURE 5:**
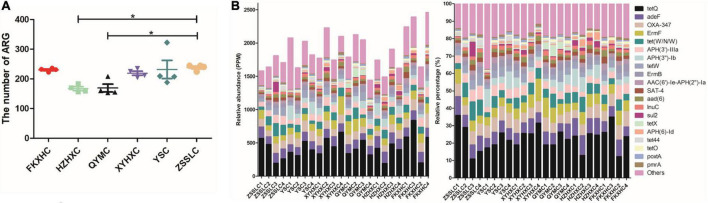
The abundance of antibiotic resistant gene. **(A)** Scatter dot plot showing the difference in the number of ARGs among the six groups. **(B)** The relative abundance in unit PPM of magnifying 10^6^ times of original abundance and the percentage of ARGs in each sample.

**FIGURE 6:**
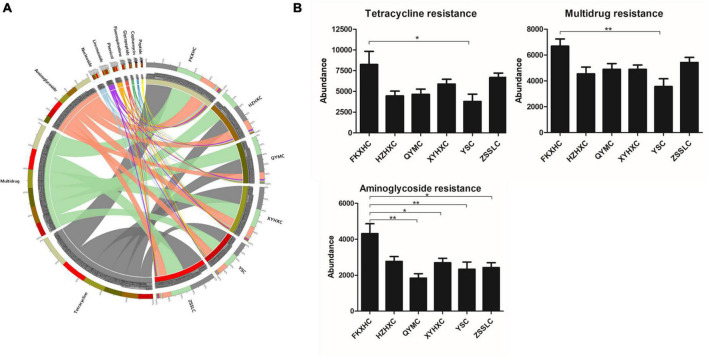
The abundance of antibiotic resistant types. **(A)** Circos plot of relative abundance of ARG types, right side of the circle indicates group information, left side indicates antibiotic information, outer circle is the ideogram scale of distribution of unique genes, and inner circle represents different groups. **(B)** Histogram showing the difference abundance in the resistance types among the six groups. Data are expressed as means ± SEM, *p < 0.05; **p < 0.01.

We identified 989 ARGs in all samples (Supplementary Table 4) using annotation results that were compared to CARD. There were some differences in ARG numbers among the six breeds of broilers. In particular, the number of ARGs in the HZXHC and QYMC groups was lower than that in the other groups (Figure 5A). The relative abundance of the top 20 ARGs is displayed as a histogram (Figure 5B); the ARG abundance in ppm and relative percentage in each broiler was calculated. The results showed that the abundance and percentage of *tet(Q)* were higher than those of other ARGs in all samples. The identified ARGs were further categorised on the basis of their resistance profiles, and each ARG was annotated with information on resistance type. The overall presence of these resistance types is depicted as a Circos plot (Figure 6A), which indicates that the predominant types of ARGs in the six groups of chickens were tetracycline, multidrug, and aminoglycoside. Additionally, we found that the FKXHC group had the highest proportion of resistance types among the six groups (Supplementary Table 4). Interestingly, the highest abundance of ARG types in the XYHXC group was also observed upon comparing the dominant types of ARGs among the six broiler groups (Figure 6B). The results imply that FKXHC have a higher risk of antibiotic resistance than the other five yellow-feathered broilers.

### 409 Draft microbial genomes assembled from yellow-feathered chicken caeca

**FIGURE 7:**
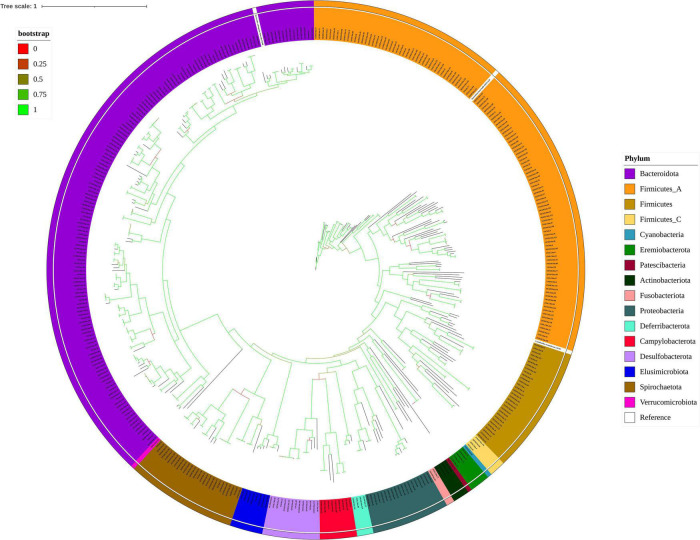
Phylogenetic tree of the 404 draft bacterial genomes from the Chinese indigenous yellow-feathered chicken caeca and three reference genomes. The taxonomies of the bins were assigned by GTDB-Tk. The different coloured ranges represent the different phylum.

In the final party of our analysis, we reconstructed genomes by co-assembly and binning of metagenomic sequencing data from 24 yellow-feathered chickens. Here, we present 409 dereplicated metagenomes (95% ANI) co-assembled bins with less than 10% contamination and greater than 80% completeness. These reconstructed genomes are described as the gut microbiomes of the Chinese yellow-feathered indigenous chickens. The total sequence length of all bins was 849 Mb. The genome sizes of individual bins ranged from 0.8 to 4.6 Mb, with N50 contigs ranging from 3.7 to 271.9 kb. The average GC content of the bins ranged from 26.2 to 66.9%. Among these genome bins, 33 bins were estimated to be near-complete genomes with ≥90% completeness and ≤5% contamination (see details in Supplementary Table 5). Next, we performed taxonomic clade research of the individual bins using GTDB which enables the objective classification of bacterial genomes assembled from the metagenomic datasets. Figure 7 shows that the phylogenetic tree of 404 bacterial bins (the tree in newick format shown in Supplementary Table 6) clearly exhibited the genomes and was dominated by two phyla: Bacteroidete (*n* = 153) and Firmicutes_A (*n* = 121), accounting for almost 70% of all bins, following by Firmicutes (*n* = 31), Spirochaetota (*n* = 27), Proteobacteria (*n* = 19), Desulfobacterota (*n* = 14), Campylobacterota (*n* = 8), Elusimicrobiota (*n* = 8), Eremiobacterota (*n* = 5), Actinobacteriota (*n* = 4), Deferribacterota (*n* = 4), Firmicutes_C (*n* = 4), Fusobacteriota (*n* = 2), Campylobacterota (*n* = 1), Cyanobacteria (*n* = 1), Patescibacteria (*n* = 1), Verrucomicrobiota (*n* = 1). We also assembled 5 archaeal genomes, which were affiliated to with three phyla: two genomes belonging to the Methanomethylophilaceae family within the Thermoplasmatota phylum, two genomes belonging to *Methanocorpusculum faecipullorum* species within the Halobacteriota phylum, and one belonging to *Methanobrevibacter_A woesei* species within the Methanobacteriota phylum (Supplementary Table 7). In addition, we contrasted with bins assembled in a recent article which comprises the largest integrated metagenomic dataset from the chicken gut to date and demonstrates the value in exploring chicken gut microbial genes (Feng et al., 2021). We found that a total of 60 species-level bins were candidate novel species, and a total of 6 genera were putative candidate novel genera (Supplementary Tables 7, 8).

## Discussion

Yellow-feathered broilers are indigenous to China. They are traditional, nutritional, and commercial mainstays for millions of people in China. Several studies have highlighted the importance of the gut microbiome in broiler health and performance. The animal intestinal microflora harbours a vast reservoir of ARGs that, when exposed and spread, may cause ecological damage (Xiong et al., 2018). Approximately 85–99% of intestinal microbes cannot be cultivated in the laboratory, which limits our understanding of bacterial functions, including those associated with antibiotic resistance (Lok, 2015). The 16S rRNA gene sequencing technology makes it difficult to decipher the functional capabilities of microbes when focussing only on limited segments of a gene (Kröber et al., 2009). Therefore, we used a metagenomic-based method to investigate differences in the gut microbial community structure of indigenous broiler breeds and reconstruct draft microbial genomes using binning. We also examined antibiotic resistance, ARGs, and their association with the host microbiome.

The caecal microbiome plays a vital role in the digestion of dietary crude fibre and affects nutrient digestion and absorption in broilers (Rodrigues et al., 2020). Therefore, the caecal microbiome has been extensively studied. In the present study, we performed a microbial metagenome analysis and reconstructed the draft microbial genomes of indigenous chicken breeds from China. The results showed that Bacteroidetes and Firmicutes were the most abundant microbes in indigenous yellow-feathered chicken, which is consistent with the results of previous studies (Mancabelli et al., 2016; Pandit et al., 2018), but the proportions of Bacteroidetes and Firmicutes were not similar, with Bacteroidetes commonly dominant. The Firmicutes/Bacteroidetes ratio of was positively correlated with feed conversion ratio (Huang et al., 2021; Lopes et al., 2021), which may be the reason for the low feed conversion ratio of indigenous yellow-feathered chickens (Deng et al., 2022). Members of these two phyla can produce short-chain fatty acids in the gut and are commonly associated with human obesity (Mariat et al., 2009), this may be responsible for the high rate of abdominal fat in yellow-feather broilers. Therefore, they play an important role in the regulation of host energy metabolism (Murugesan et al., 2015). At the genus level, we found that the most dominant genus in the indigenous chicken was *Bacteroides*, which plays an important role in decomposing polysaccharides that are useful to animal hosts (Huang et al., 2021). Furthermore, the major dominant genera in QYMC were *Prevotella* and *Bacteroides*, and the relative abundance of *Prevotella* was significantly higher than that of the other five groups. *Prevotella* abundance has been shown to be important for carbohydrate utilisation and can increase host fat accumulation (Chen et al., 2021). Tan et al. (2017) also found a high relative abundance of *Prevotella* in pigs with both low and high feed efficiency. Gut microbiomes are closely related to chicken performance, and studies have found that feed supplemented with *Lactobacillus* can increase chicken body weight (Angelakis and Raoult, 2010). The abundance of *Bacteroides* and *Bifidobacterium* in the ileum was positively correlated with body weight (Han et al., 2016), where *Bacteroides* may be utilised as a biomaker for feed efficiency (Huang et al., 2021; Dittoe et al., 2022). Understanding the gut microbiome of yellow-feathered chickens contributes to the success of poultry meat production, which requires improved feed efficiency, increased muscle mass, and reduced abdominal fat ratio (Zerehdaran et al., 2004). The PCoA and NMDS plots showed a clear population structure among samples of the different lines in yellow-feathered broilers, particularly in the HZHXC, FKXHC, ZSSLC, and QYMC. A similar pattern has been reported in previous studies (Miller et al., 2021). Several factors, such as breed, age, and feed, may cause variations in the gut microbiota (Wang et al., 2019). In addition, previous studies have revealed the significant influence of host genetics and environmental factors on the composition of the gut microbiome (Crespo-Piazuelo et al., 2019). An example is the study by Goodrich et al. (2017) who found evidence that the host genome influences the composition of microbiome in humans. Metabolism, genetic information processing, cellular processes, human diseases, and organismal systems were the dominant functions predicted at level one in our KEGG pathway analysis. The caecal microbiota of yellow-feathered chicken has high abundance of functions involved in metabolic pathway for carbohydrate, amino acid, and nucleotide metabolism. Utilisation of carbohydrates is important in chicken growth. Amino metabolism specifically is responsible for breaking down protein present in feed to peptides and amino acids (Miska et al., 2014). Nucleotide metabolism is important for the synthesis of purines and pyrimidines, which are important substrates for deoxyribonucleic acid derivatives (Lane and Fan, 2015). Regarding carbohydrate-active enzymes encoding genes, we found GHs are major enzymes in the chicken gut microbiome. As starch from the cornmeal was the major source of carbohydrates in the feed of the chicken and GHs catalyses the hydrolysis of glycosidic bonds reported in complex carbohydrates, including starch (Vu and Marletta, 2016; Maia et al., 2021), it was not surprising to see a high potential for digestion, with many genes encoding GHs.

In the current study, metagenomic sequencing was used to identify the presence and relative abundance of ARGs in yellow-feathered broilers. The results showed that 989 ARGs were present, including *tetQ*, *adeF*, *OXA-34*7, *Ermf*, and *tetX*. The *tetQ* gene had the highest relative abundance in all the samples. *TetQ* is a ribosomal protection protein that is associated with a conjugative transposon and has been found in both gramme-positive and gramme-negative bacteria. Next, we matched each ARG to its corresponding antibiotic and concluded that the ARGs in the chicken gut microbiota conferred resistance to almost all the major antibiotic classes commonly employed for agricultural use. In the yellow-feathered chickens, tetracycline, multidrug, and aminoglycoside resistance was most commonly found. In recent decades, tetracyclines have been widely incorporated into animal feed to reduce the incidence of diseases and increase growth rates. Although China banned the use of antibiotics as growth promoters in 2020, ARGs have already been deposited in microbial genomes (Yang et al., 2022). All yellow feather broilers also did not receive any antibiotic treatment in our experiment, and the observation that the broiler gut microbiome has a broad-ranging resistome is not surprising. Rovira et al. (2019) found that discontinuing the use of tetracyclines in farms did not cause an automatic reduction in resistance. Ma et al. (2016) reported that ARGs can be transmitted from livestock animals to humans and are widely spread across different environments. Hence, careful use of antibiotics is recommended to prevent the increase and transfer of key ARGs from farms. Notably, *tetX* was detected in the chicken intestine. Although tigecycline has not been used in animal husbandry, *tetX* was still found in the intestinal microbiome of chickens in this study, presumably due to the selection pressure of tetracycline. Notably, *tetX* was detected in the chicken intestine. Although tigecycline has not been used in animal husbandry, *tetX* was still found in the intestinal microbiome of chickens in this study, presumably due to the selection pressure of tetracycline. The recent emergence of plasmid-mediated high levels of tigecycline resistance genes *tetX3* and *tetX4* from animals and humans has triggered the much broader public alarm (He et al., 2019). Therefore, it is important to improve the robust monitoring of *tetX* genes. We found that the FKXHC had a higher abundance of resistance genes than the other five breeds of yellow-feathered chickens. The factors influencing the high abundance of ARGs in FKXHC have not been fully explained yet. Fodder is a potential source of ARGs in chickens that undergo dynamic changes in their feed composition. The farm environment also serves as a potential source of resistant bacteria that can become resident in the chicken gut (Zalewska et al., 2021). However, given the complexity of the sources of ARGs, it is difficult to explain the drivers of increased resistance in the gut of FKXHC. The composition of ARG types would be different because of the distinct microbial community structure and diversity of both faeces and the internal environment of the chicken gut (Huang et al., 2018), or different feeding patterns of different broiler breeds (Jochum et al., 2021). In summary, the results showed that the gut of commercial broilers contained drug-resistant bacteria. Despite great efforts to reduce contamination during the slaughter process, resistant and pathogenic bacteria in faeces may cause potential pollution and public health risks. Additional risks may exist because of environmental pollution ordirect exposure of farm labourers.

In summary, the reconstruction of 409 nearly complete draft genomes from the caecal microbiome of Chinese indigenous yellow-feathered broilers, represented a broad microbial population from 16 bacterial phyla and 3 archaeal phyla, and we found that 60 and 6 bins in our dataset were putative novel species and genera, respectively. Bacteroidetes and Firmicutes were the dominant phyla, *Bacteroides* was the dominant genus, and the main antibiotic-resistant types were tetracycline, multidrug, and aminoglycoside. In addition, we found that the relative abundance of *Prevotella* in QYMC was significantly higher than that in the other five breeds, and FKXHC might have a higher risk of resistance to antibiotic treatment than the other five yellow-feathered broilers. To our knowledge, this study is the first to provide comprehensive microbiome genomes and antibiotic-resistance profiles for Chinese yellow-feathered broilers, thereby providing a useful indicator for the use of antibacterial products in yellow-feathered broilers.

## Data availability statement

The datasets presented in this study can be found in online repositories. The names of the repository/repositories and accession number(s) can be found below: Genome Sequence Archive (GSA), GSA number: CRAA006801.

## Ethics statement

The animal study was reviewed and approved by the Institutional Animal Care and Use Committee of the South China Agricultural University.

## Author contributions

YX participated in the design of the experiment and wrote the manuscript and data analyses. HX participated in data analyses, engaged in useful discussion, and revised the manuscript. YH and LG participated in data analyses. SZ, RW, and XF participated in sample collection. QN developed the concepts, designed and supervised the study, and revised the manuscript. All authors contributed to the article and approved the submitted version.

## Footnotes

1
http://exon.gatech.edu/meta_gmhmmp.cgi

2
http://github.com/dparks1134/CompareM

3
www.biocloud.net
