# Supplementary material for: Metagenomic analysis reveals the microbiome and antibiotic resistance genes in indigenous Chinese yellow-feathered chickens
Source: Front Microbiol. 2022 Sep 7;13:930289. doi: 10.3389/fmicb.2022.930289 (PMC9490229; doi:10.3389/fmicb.2022.930289)
Supplement: Supplementary file 6 [file Table_6.docx]

**Supplemental table 8: The newick format of phylogenetic tree**

(XYHXCbin.73:0.0079597,(QYMCbin.73:0.014397275,((HZHXCbin.70:0.072520549,HZHXCbin.5:0.040668153):0.012875017[0.869],((ZSSLCbin.67:0.000263115,HZHXCbin.18:0.004256056):0.044673391[1.000],(((ZSSLCbin.7:0.005947780,HZHXCbin.68:0.000331972):0.078417845[1.000],(HZHXCbin.12:0.002136172,(XYHXCbin.74:0.000931802,QYMCbin.60:0.003015490):0.004464628[0.862]):0.074936732[1.000]):0.046805674[1.000],(((YSCbin.56:0.006089740,HZHXCbin.38:0.001387301):0.204346139[1.000],(HZHXCbin.90:0.150991647,((XYHXCbin.166:0.001820733,HZHXCbin.64:0.010319533):0.024570989[1.000],(QYMCbin.114:0.000841502,FKXHCbin.44:0.003916283):0.029489660[1.000]):0.113782589[1.000]):0.043353072[1.000]):0.035386732[1.000],((XYHXCbin.175:0.009142785,((XYHXCbin.167:0.000732944,FKXHCbin.63:0.000649544):0.000690602[0.788],(ZSSLCbin.87:0.000000005,(HZHXCbin.47:0.001758104,YSCbin.107:0.000880842):0.000208884[0.131]):0.000632940[0.672]):0.012515644[0.999]):0.143306647[1.000],((ZSSLCbin.51:0.000619231,YSCbin.61:0.005288314):0.207047197[1.000],(((YSCbin.100:0.000646795,(XYHXCbin.71:0.000466017,(ZSSLCbin.102:0.000859205,FKXHCbin.29:0.001014076):0.000216325[0.748]):0.000265165[0.748]):0.194748572[1.000],((QYMCbin.112:0.000477375,((XYHXCbin.84:0.000708293,YSCbin.136:0.000981737):0.000439412[0.880],(HZHXCbin.52:0.001607239,(ZSSLCbin.154:0.001135969,FKXHCbin.24:0.001128559):0.000000005[0.761]):0.000218838[0.753]):0.000222989[0.267]):0.145473752[1.000],((ZSSLCbin.124:0.000558835,YSCbin.35:0.000000005):0.111431532[1.000],((HZHXCbin.116:0.085561182,(QYMCbin.95:0.000814402,(ZSSLCbin.103:0.000000005,HZHXCbin.56:0.000654205):0.000276586[0.758]):0.092976868[1.000]):0.040532375[1.000],(QYMCbin.47:0.052175828,((XYHXCbin.126:0.000937355,HZHXCbin.126:0.000305781):0.000452389[0.752],(YSCbin.77:0.000617885,ZSSLCbin.164:0.010894599):0.000274673[0.279]):0.030542633[1.000]):0.081462197[1.000]):0.032221359[1.000]):0.026099969[0.997]):0.044539741[1.000]):0.029524552[1.000],((QYMCbin.124:0.212154351,Anaerobutyricumbin.1:0.228176163):0.041491221[0.997],((HZHXCbin.125:0.127112675,((HZHXCbin.15:0.062485563,HZHXCbin.128:0.065988200):0.105816838[1.000],(YSCbin.119:0.001909911,HZHXCbin.109:0.003377842):0.191247560[1.000]):0.059452385[1.000]):0.142501801[1.000],((FKXHCbin.54:0.002075079,(YSCbin.72:0.000805635,(HZHXCbin.120:0.000339813,ZSSLCbin.78:0.000964763):0.000864925[0.849]):0.000097967[0.446]):0.313239466[1.000],((FKXHCbin.48:0.521718720,((ZSSLCbin.149:0.348997760,(ZSSLCbin.80:0.003226508,QYMCbin.113:0.003456568):0.314426039[1.000]):0.142706848[1.000],(((ZSSLCbin.70:0.223112049,(ZSSLCbin.144:0.246187803,(ZSSLCbin.136:0.001175833,YSCbin.109:0.003131956):0.272312912[1.000]):0.054656696[0.994]):0.105081858[1.000],(FKXHCbin.31:0.296774370,((QYMCbin.145:0.226913607,(XYHXCbin.148:0.176445626,HZHXCbin.92:0.204512031):0.120779489[1.000]):0.042666510[0.752],(YSCbin.44:0.154498969,(YSCbin.150:0.097384913,(YSCbin.26:0.080887026,(YSCbin.76:0.004788279,FKXHCbin.43:0.006426311):0.054687880[1.000]):0.063616305[1.000]):0.068465800[1.000]):0.179375462[1.000]):0.079280233[1.000]):0.049416629[0.221]):0.066642085[1.000],(XYHXCbin.106:0.403918691,((FKXHCbin.148:0.292461955,(ZSSLCbin.23:0.153160420,(YSCbin.70:0.015778231,HZHXCbin.75:0.031333900):0.186154144[1.000]):0.212930910[1.000]):0.052853979[0.980],(QYMCbin.40:0.294301335,((QYMCbin.27:0.259574741,(ZSSLCbin.19:0.166341017,ZSSLCbin.133:0.215156156):0.057519897[0.996]):0.060964537[1.000],(((XYHXCbin.60:0.000000005,FKXHCbin.126:0.001184041):0.163147286[1.000],((FKXHCbin.32:0.003119496,HZHXCbin.123:0.002212209):0.001471641[0.437],(XYHXCbin.31:0.006685570,ZSSLCbin.49:0.001980122):0.002744677[0.651]):0.149392315[1.000]):0.116219500[1.000],(QYMCbin.109:0.169981639,((YSCbin.9:0.074370887,XYHXCbin.39:0.073327628):0.069977434[1.000],(XYHXCbin.121:0.080270624,(QYMCbin.99:0.034124993,(YSCbin.6:0.001858678,HZHXCbin.105:0.000892511):0.043977165[1.000]):0.027456510[1.000]):0.090314783[1.000]):0.049196725[1.000]):0.123838081[1.000]):0.069264109[1.000]):0.040302310[0.051]):0.040107578[0.996]):0.055316415[1.000]):0.029458619[0.180]):0.041177894[0.998]):0.083867606[1.000]):0.036202933[0.993],((((ZSSLCbin.6:0.280482971,ZSSLCbin.41:0.300393337):0.097315660[1.000],((XYHXCbin.35:0.054048422,FKXHCbin.66:0.048941739):0.187695229[1.000],(YSCbin.120:0.001905784,FKXHCbin.77:0.002681099):0.220490841[1.000]):0.164149756[1.000]):0.088819015[1.000],(QYMCbin.107:0.582254965,((ZSSLCbin.17:0.440123549,(XYHXCbin.4:0.257994613,QYMCbin.131:0.250464409):0.212326125[1.000]):0.048529147[0.545],(((FKXHCbin.114:0.054749157,(ZSSLCbin.95:0.003476960,QYMCbin.150:0.002900724):0.042744791[1.000]):0.332649491[1.000],(FKXHCbin.80:0.003616594,(ZSSLCbin.38:0.003017553,XYHXCbin.143:0.003967576):0.002212099[0.859]):0.398909736[1.000]):0.057791689[0.879],(ZSSLCbin.73:0.259329599,((ZSSLCbin.127:0.184197224,(ZSSLCbin.110:0.002991059,QYMCbin.128:0.004763890):0.210776660[1.000]):0.083317607[1.000],(ZSSLCbin.114:0.000736454,(XYHXCbin.15:0.001659395,FKXHCbin.12:0.001850149):0.000722691[0.812]):0.245906404[1.000]):0.041524853[0.008]):0.141596460[1.000]):0.057293116[1.000]):0.048428796[0.983]):0.126928037[1.000]):0.071388951[1.000],((ZSSLCbin.61:0.204790360,FKXHCbin.103:0.224466068):0.266503992[1.000],((((Lactobacillusbin.1:0.093739153,FKXHCbin.72:0.080226278):0.270766505[1.000],(ZSSLCbin.30:0.179180949,(YSCbin.101:0.103558276,(ZSSLCbin.123:0.060888186,YSCbin.33:0.045396753):0.086599277[1.000]):0.119873024[1.000]):0.086954105[1.000]):0.206601345[1.000],((QYMCbin.96:0.306974576,(QYMCbin.13:0.315718804,((XYHXCbin.107:0.163254737,(YSCbin.38:0.000855446,FKXHCbin.176:0.001832408):0.279745314[1.000]):0.114775922[1.000],(QYMCbin.43:0.247260563,(XYHXCbin.36:0.005310521,(ZSSLCbin.1:0.000857688,QYMCbin.76:0.003149243):0.001063924[0.722]):0.247103538[1.000]):0.061680306[0.999]):0.073490964[1.000]):0.058851293[0.813]):0.200986938[1.000],(((HZHXCbin.127:0.171637226,FKXHCbin.76:0.168626766):0.231360866[1.000],(XYHXCbin.162:0.381675833,(FKXHCbin.123:0.257151394,(FKXHCbin.140:0.078417847,(XYHXCbin.69:0.001726981,FKXHCbin.35:0.001402902):0.082040853[1.000]):0.128649772[1.000]):0.110161483[1.000]):0.119553041[1.000]):0.069242783[0.999],((QYMCbin.98:0.001767138,HZHXCbin.13:0.001281760):0.674894020[1.000],((XYHXCbin.20:0.002015528,FKXHCbin.116:0.002641900):0.362529129[1.000],(FKXHCbin.178:0.421263915,((QYMCbin.18:0.385447460,FKXHCbin.169:0.309738547):0.064364855[0.932],(QYMCbin.115:0.324213311,(XYHXCbin.33:0.009841431,FKXHCbin.19:0.002436229):0.278788596[1.000]):0.210635950[1.000]):0.037069350[0.340]):0.077522555[1.000]):0.063680461[0.789]):0.156626061[1.000]):0.068151502[1.000]):0.084104540[1.000]):0.130514457[1.000],((XYHXCbin.178:0.303892049,(QYMCbin.66:0.001174788,(ZSSLCbin.4:0.001040701,YSCbin.93:0.006167246):0.000000008[0.365]):0.329763289[1.000]):0.163504606[1.000],((XYHXCbin.83:0.643331586,(YSCbin.142:0.553550077,(HZHXCbin.97:0.000218081,(FKXHCbin.113:0.000219331,(YSCbin.130:0.000438757,XYHXCbin.151:0.000000005):0.000000005[0.000]):0.000000005[0.000]):0.544938275[1.000]):0.164780162[1.000]):0.059510077[0.914],((FKXHCbin.173:0.996525958,((FKXHCbin.69:0.231703999,FKXHCbin.100:0.200766773):0.368529998[1.000],(XYHXCbin.25:0.237686544,QYMCbin.157:0.205520477):0.493604817[1.000]):0.081508629[0.947]):0.068177614[0.999],((ZSSLCbin.71:0.000969209,FKXHCbin.97:0.000570587):0.684099987[1.000],((((FKXHCbin.45:0.471355878,(QYMCbin.88:0.051020061,((ZSSLCbin.64:0.003815123,QYMCbin.122:0.004785298):0.059354211[1.000],(YSCbin.80:0.049962589,(ZSSLCbin.65:0.015272106,HZHXCbin.99:0.010217438):0.044214024[1.000]):0.030144239[1.000]):0.029278186[0.986]):0.554215459[1.000]):0.231686751[1.000],((ZSSLCbin.113:0.160007454,(ZSSLCbin.105:0.185636444,(ZSSLCbin.18:0.121867658,HZHXCbin.26:0.129327759):0.081763426[1.000]):0.040718168[0.737]):0.293071980[1.000],(FKXHCbin.84:0.232443282,((HZHXCbin.19:0.000000005,(ZSSLCbin.36:0.001569524,XYHXCbin.29:0.001041325):0.000399773[0.036]):0.123474549[1.000],(XYHXCbin.7:0.098845389,(XYHXCbin.26:0.000649338,(HZHXCbin.45:0.001071577,FKXHCbin.96:0.001013920):0.001573404[0.896]):0.085498500[1.000]):0.102360202[1.000]):0.245139290[1.000]):0.131941005[1.000]):0.176466611[1.000]):0.086619663[1.000],((QYMCbin.137:0.061082900,(FKXHCbin.154:0.031950714,(ZSSLCbin.86:0.000715870,QYMCbin.134:0.001930687):0.033191788[1.000]):0.046223313[1.000]):0.621986229[1.000],((((YSCbin.52:0.000596676,FKXHCbin.49:0.002469917):0.087995967[1.000],(QYMCbin.32:0.002433930,HZHXCbin.30:0.002516490):0.091164111[1.000]):0.298156753[1.000],((XYHXCbin.180:0.266230276,FKXHCbin.62:0.243548435):0.090717323[1.000],(YSCbin.10:0.001219452,(ZSSLCbin.108:0.000730106,HZHXCbin.133:0.001388515):0.000349801[0.735]):0.237082937[1.000]):0.115359464[1.000]):0.517108949[1.000],(ZSSLCbin.46:0.294159909,((XYHXCbin.174:0.099850545,(YSCbin.138:0.086708562,(QYMCbin.44:0.000559887,(ZSSLCbin.93:0.002736899,FKXHCbin.186:0.000420031):0.000922142[0.810]):0.117096861[1.000]):0.025385845[0.011]):0.069917231[1.000],((YSCbin.49:0.000577831,QYMCbin.93:0.002143319):0.088050064[1.000],((HZHXCbin.121:0.006589502,XYHXCbin.157:0.001041808):0.000301000[0.103],(YSCbin.54:0.000745112,(QYMCbin.79:0.001792696,(ZSSLCbin.66:0.002715275,FKXHCbin.23:0.001397877):0.000482092[0.058]):0.000361704[0.736]):0.000182838[0.692]):0.090317562[1.000]):0.187559221[1.000]):0.111919186[1.000]):0.374250024[1.000]):0.056637406[0.164]):0.044260206[0.673]):0.050338750[1.000],(((HZHXCbin.102:0.084776444,(XYHXCbin.32:0.027367723,(ZSSLCbin.81:0.018675728,QYMCbin.7:0.023755869):0.018580063[0.998]):0.069758930[1.000]):0.047942833[0.996],(XYHXCbin.146:0.059267089,(HZHXCbin.114:0.002154813,(ZSSLCbin.160:0.002947927,YSCbin.17:0.001042610):0.000976449[0.470]):0.031372191[1.000]):0.078887320[1.000]):0.714143411[1.000],((((XYHXCbin.2:0.268825271,(YSCbin.63:0.000000005,(HZHXCbin.7:0.002033299,XYHXCbin.103:0.001582909):0.000942410[0.771]):0.205352285[1.000]):0.053109053[0.525],(XYHXCbin.149:0.004211650,(QYMCbin.61:0.002801470,(YSCbin.79:0.001376399,FKXHCbin.177:0.001534741):0.000824719[0.933]):0.000215449[0.650]):0.255050104[1.000]):0.196892647[1.000],((FKXHCbin.182:0.000985237,((XYHXCbin.18:0.001509218,HZHXCbin.35:0.001520583):0.000213721[0.730],(QYMCbin.57:0.001989958,YSCbin.148:0.002424919):0.000428928[0.877]):0.000000005[0.632]):0.463286814[1.000],(((QYMCbin.39:0.049223463,QYMCbin.123:0.056732625):0.171038164[1.000],(QYMCbin.138:0.129967496,(XYHXCbin.80:0.001562523,(QYMCbin.52:0.000682732,FKXHCbin.138:0.002402431):0.001385983[0.811]):0.110792603[1.000]):0.081033716[1.000]):0.070578239[1.000],((XYHXCbin.99:0.003507169,FKXHCbin.20:0.002291378):0.084531020[1.000],((FKXHCbin.164:0.095662069,(XYHXCbin.57:0.001598781,FKXHCbin.104:0.001451347):0.072311247[1.000]):0.020890125[0.365],(HZHXCbin.29:0.001291224,(QYMCbin.62:0.002563281,YSCbin.8:0.002274127):0.001606843[0.944]):0.081067929[1.000]):0.034215382[0.997]):0.130349713[1.000]):0.244236153[1.000]):0.063364390[0.176]):0.361163391[1.000],(ZSSLCbin.134:0.773103015,(((FKXHCbin.111:0.278806101,(YSCbin.11:0.022978647,HZHXCbin.130:0.022304391):0.288144931[1.000]):0.055165720[0.985],(YSCbin.91:0.000677491,(HZHXCbin.53:0.000623788,(XYHXCbin.27:0.000679875,FKXHCbin.42:0.001462971):0.000600671[0.796]):0.000320916[0.780]):0.317826054[1.000]):0.071654296[1.000],(((FKXHCbin.47:0.168734959,((ZSSLCbin.29:0.145869183,(ZSSLCbin.40:0.001376088,YSCbin.124:0.001938830):0.161219118[1.000]):0.029954046[0.971],(ZSSLCbin.58:0.125913870,((ZSSLCbin.34:0.030217973,(QYMCbin.132:0.000996927,(ZSSLCbin.137:0.002651001,(YSCbin.3:0.001238662,(XYHXCbin.111:0.008883525,HZHXCbin.43:0.002954318):0.000000011[0.807]):0.000239828[0.750]):0.000947462[0.414]):0.021961665[1.000]):0.087331786[1.000],((YSCbin.27:0.158558315,ZSSLCbin.74:0.072456995):0.030894507[0.996],(YSCbin.74:0.062274431,(ZSSLCbin.25:0.016362149,(XYHXCbin.41:0.001210135,(HZHXCbin.57:0.000444219,QYMCbin.140:0.000217154):0.001175756[0.925]):0.017233459[1.000]):0.039117581[1.000]):0.042012799[1.000]):0.021696626[0.199]):0.055294668[1.000]):0.062809269[1.000]):0.033699744[0.995]):0.133565809[1.000],(((ZSSLCbin.92:0.117245246,(XYHXCbin.105:0.000000005,FKXHCbin.184:0.001629372):0.126127798[1.000]):0.109023342[1.000],((HZHXCbin.49:0.000000005,(XYHXCbin.104:0.001403521,YSCbin.42:0.001172726):0.000493202[0.901]):0.045441487[1.000],(XYHXCbin.165:0.000296773,(YSCbin.135:0.000956062,QYMCbin.147:0.001942614):0.000278795[0.447]):0.053783517[1.000]):0.157002908[1.000]):0.029862228[0.139],(((YSCbin.25:0.001423548,(XYHXCbin.17:0.002063825,HZHXCbin.95:0.002869080):0.002387378[0.954]):0.064769238[1.000],((XYHXCbin.5:0.000305018,FKXHCbin.150:0.000730069):0.060245992[1.000],(YSCbin.106:0.004527104,(QYMCbin.117:0.002876474,HZHXCbin.91:0.000694231):0.003186612[0.880]):0.049169356[1.000]):0.030016351[1.000]):0.121818282[1.000],((QYMCbin.151:0.039558657,(QYMCbin.87:0.002619468,(YSCbin.90:0.002766124,HZHXCbin.93:0.001909731):0.001744774[0.810]):0.033225847[1.000]):0.213164916[1.000],(((XYHXCbin.16:0.037272114,(YSCbin.143:0.005931796,HZHXCbin.20:0.002227547):0.034172942[1.000]):0.027414642[1.000],(XYHXCbin.145:0.000523638,(HZHXCbin.66:0.000223516,YSCbin.58:0.000000005):0.000472700[0.856]):0.041496866[1.000]):0.056287287[1.000],(XYHXCbin.24:0.116258005,((YSCbin.105:0.053034931,(XYHXCbin.38:0.058308546,(YSCbin.60:0.000813020,XYHXCbin.135:0.000000005):0.039352293[1.000]):0.011793399[0.416]):0.036469729[1.000],((YSCbin.149:0.001420848,XYHXCbin.116:0.002405352):0.043085691[1.000],(QYMCbin.75:0.030066661,(YSCbin.92:0.001876787,FKXHCbin.53:0.000271893):0.027816517[1.000]):0.018815063[1.000]):0.042350492[1.000]):0.016191203[0.936]):0.016083374[0.758]):0.033196092[0.998]):0.063739555[1.000]):0.064293460[1.000]):0.135461257[1.000]):0.049792264[1.000],(((QYMCbin.8:0.063448903,ZSSLCbin.111:0.052910899):0.082324089[1.000],(QYMCbin.105:0.000374532,(HZHXCbin.131:0.002101516,(FKXHCbin.3:0.000389737,(YSCbin.83:0.000958564,ZSSLCbin.163:0.002692939):0.000402679[0.405]):0.000256441[0.693]):0.000299080[0.455]):0.127098713[1.000]):0.180535824[1.000],(((XYHXCbin.90:0.005615300,FKXHCbin.118:0.001420026):0.265086169[1.000],(XYHXCbin.43:0.003934168,(FKXHCbin.79:0.003138273,HZHXCbin.138:0.003542159):0.002124054[0.258]):0.201135859[1.000]):0.037285506[0.861],((ZSSLCbin.84:0.125777771,(((ZSSLCbin.153:0.024504009,XYHXCbin.120:0.031462000):0.098906483[1.000],(YSCbin.82:0.000222439,XYHXCbin.70:0.001657106):0.199141239[1.000]):0.022595211[0.730],(((ZSSLCbin.90:0.000959066,FKXHCbin.119:0.002109896):0.140531384[1.000],((XYHXCbin.119:0.026446580,FKXHCbin.17:0.023958696):0.010317795[0.857],(ZSSLCbin.77:0.024701558,ZSSLCbin.131:0.026201824):0.011446540[0.971]):0.081525188[1.000]):0.034585869[0.997],(ZSSLCbin.47:0.216588086,(((HZHXCbin.79:0.000404732,(XYHXCbin.113:0.000000005,QYMCbin.156:0.000000005):0.000612813[0.848]):0.072857917[1.000],((HZHXCbin.98:0.024275538,HZHXCbin.101:0.023002842):0.025267065[1.000],(QYMCbin.143:0.001733440,HZHXCbin.65:0.000877812):0.052458422[1.000]):0.039604074[1.000]):0.094005772[1.000],(FKXHCbin.188:0.080399083,(((XYHXCbin.100:0.004293414,QYMCbin.1:0.003232045):0.035319151[1.000],(YSCbin.71:0.002049873,XYHXCbin.68:0.003025757):0.028913480[1.000]):0.059203237[1.000],(HZHXCbin.23:0.000600224,((YSCbin.36:0.001183391,QYMCbin.58:0.001769797):0.000739870[0.930],(XYHXCbin.54:0.002072116,FKXHCbin.74:0.001683987):0.000299092[0.744]):0.000499517[0.575]):0.107605072[1.000]):0.020817010[0.925]):0.082405149[1.000]):0.028929631[0.166]):0.089203955[1.000]):0.031743214[1.000]):0.023586284[0.979]):0.052991600[1.000],((ZSSLCbin.88:0.077359420,((XYHXCbin.164:0.001427505,HZHXCbin.40:0.000584035):0.066111333[1.000],(ZSSLCbin.83:0.059748784,(ZSSLCbin.9:0.041935095,(ZSSLCbin.82:0.014655559,(YSCbin.7:0.001290628,QYMCbin.56:0.001288478):0.015456444[1.000]):0.050537326[1.000]):0.046726184[1.000]):0.018341466[0.970]):0.020031082[0.333]):0.111234426[1.000],(((HZHXCbin.24:0.002346482,(QYMCbin.65:0.002679741,(XYHXCbin.168:0.000810249,YSCbin.15:0.000000005):0.001090993[0.824]):0.000803389[0.673]):0.149508108[1.000],((YSCbin.85:0.000768324,XYHXCbin.144:0.000973204):0.093084902[1.000],((YSCbin.22:0.002648677,XYHXCbin.28:0.008183407):0.067658898[1.000],(YSCbin.41:0.001016390,FKXHCbin.87:0.001654155):0.079030428[1.000]):0.077049435[1.000]):0.045910510[1.000]):0.051113058[1.000],(((XYHXCbin.81:0.002382888,HZHXCbin.88:0.000540450):0.216028187[1.000],(((ZSSLCbin.139:0.065200680,QYMCbin.22:0.085207999):0.062356620[1.000],((YSCbin.4:0.060891186,QYMCbin.162:0.082004030):0.056120843[1.000],(YSCbin.146:0.001822733,FKXHCbin.180:0.003517406):0.119918137[1.000]):0.088522993[1.000]):0.024683671[0.358],((HZHXCbin.54:0.000742506,(QYMCbin.48:0.000000005,YSCbin.127:0.000287920):0.001817471[0.942]):0.182204159[1.000],((QYMCbin.11:0.037729518,HZHXCbin.145:0.042962068):0.063832111[1.000],(FKXHCbin.16:0.001541383,(ZSSLCbin.146:0.000517516,YSCbin.139:0.001620249):0.000000006[0.456]):0.140993821[1.000]):0.109145044[1.000]):0.034493939[0.999]):0.041220733[1.000]):0.021486442[0.241],((ZSSLCbin.159:0.099665360,(QYMCbin.42:0.034537265,Bacteroidesbin.1:0.032723091):0.027243386[1.000]):0.045617056[1.000],((XYHXCbin.63:0.000294690,(ZSSLCbin.125:0.001773329,HZHXCbin.80:0.000262675):0.000000005[0.720]):0.100585166[1.000],(((ZSSLCbin.141:0.033543051,HZHXCbin.81:0.054155192):0.018275844[0.995],(QYMCbin.24:0.044115103,(XYHXCbin.134:0.000339062,HZHXCbin.119:0.000481505):0.026241405[1.000]):0.017351166[1.000]):0.012929126[0.865],((QYMCbin.30:0.045689918,(XYHXCbin.139:0.003461402,HZHXCbin.136:0.001921962):0.076520243[1.000]):0.011996535[0.980],(XYHXCbin.142:0.034873502,(YSCbin.81:0.000699538,HZHXCbin.48:0.000367910):0.036007168[1.000]):0.026843677[1.000]):0.009980199[0.947]):0.047540101[1.000]):0.019226344[0.852]):0.025796051[1.000]):0.026739951[0.735]):0.073896545[1.000]):0.033226696[0.974]):0.047170028[1.000]):0.090508941[1.000]):0.046274550[1.000]):0.042333253[0.996]):0.392196034[1.000]):0.069324993[0.959]):0.043121249[0.903]):0.025397334[0.686]):0.045688898[0.999]):0.024810648[0.559]):0.025680115[0.732]):0.042298650[1.000]):0.029699403[0.703]):0.056830100[1.000]):0.043206822[1.000]):0.028712100[0.939]):0.124776617[1.000]):0.122329213[1.000]):0.047694590[0.465]):0.032578128[1.000]):0.023637670[0.982]):0.023572741[0.522]):0.016143200[0.348]):0.074521216[1.000]):0.063609975[1.000]):0.011333475[0.933]):0.050483319[1.000]):0.0079597);
