# Supplementary material for: Metagenomic analysis reveals the microbiome and antibiotic resistance genes in indigenous Chinese yellow-feathered chickens
Source: Front Microbiol. 2022 Sep 7;13:930289. doi: 10.3389/fmicb.2022.930289 (PMC9490229; doi:10.3389/fmicb.2022.930289)
Supplement: Supplementary file 9 [file Table_9.docx]

**supplemental figures**

**Metagenomic analysis reveals the microbiome and antibiotic resistance genes in Guangdong yellow-feathered indigenous chicken**

**Authors:**

Yibin Xu^1,2^, Lijin Guo^1,2^, Yulin Huang^1,2^, Siyu Zhang^1,2^, Xiquan Zhang^1,2^, and Qinghua Nie^1,2*^

^1^Lingnan Guangdong Laboratory of Modern Agriculture & State Key Laboratory for Conservation and Utilization of Subtropical Agro-bioresources, College of Animal Science, South China Agricultural University, Guangzhou 510642, Guangdong, China.

^2^Guangdong Provincial Key Lab of AgroAnimal Genomics and Molecular Breeding and Key Lab of Chicken Genetics, Breeding and Reproduction, Ministry of Agriculture, Guangzhou, 510642, Guangdong, China.

* Corresponding author: Qinghua Nie

Address: College of Animal Science, South China Agricultural University, Guangzhou 510642, Guangdong Province, China.

Tel: + 86 20 85285759

E-mail: nqinghua@scau.edu.cn


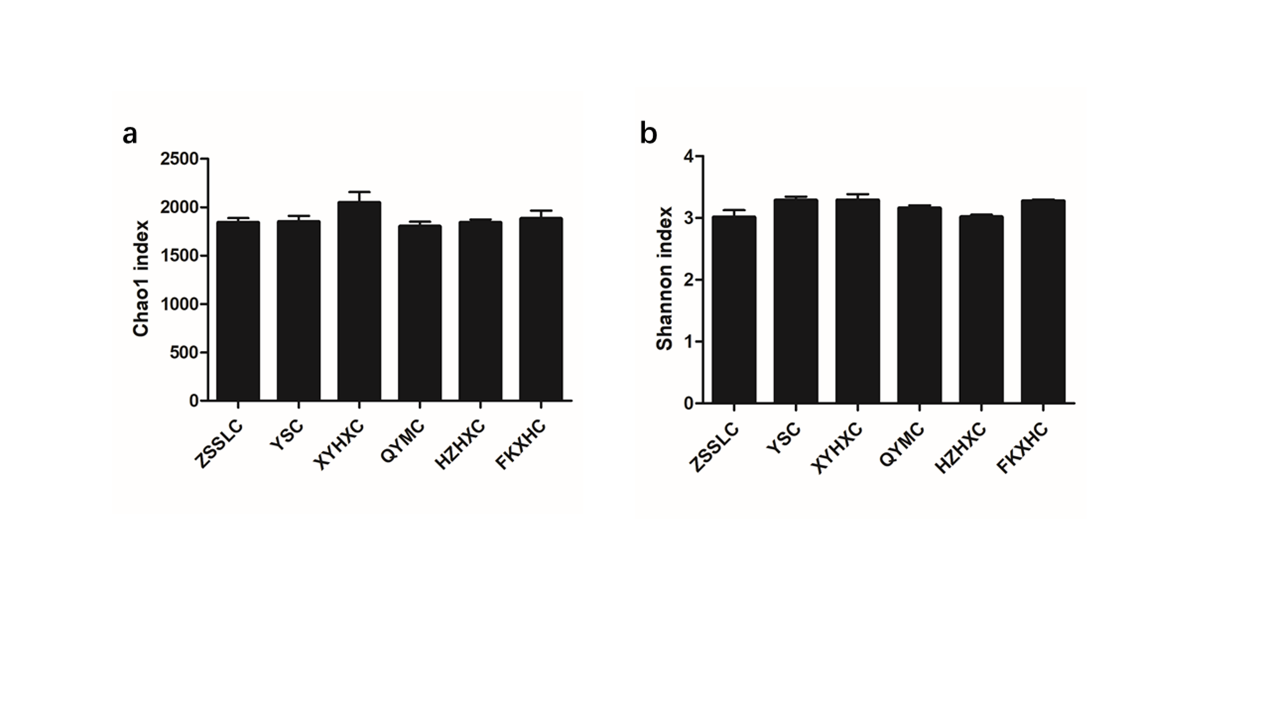


Figure S1. alpha diversity of bacterial communities **(a)** Chao1 index, **(b)** Shannon index. Data are expressed as means ± SEM, **p* < 0.05; ***p* < 0.01; ****p* < 0.001.


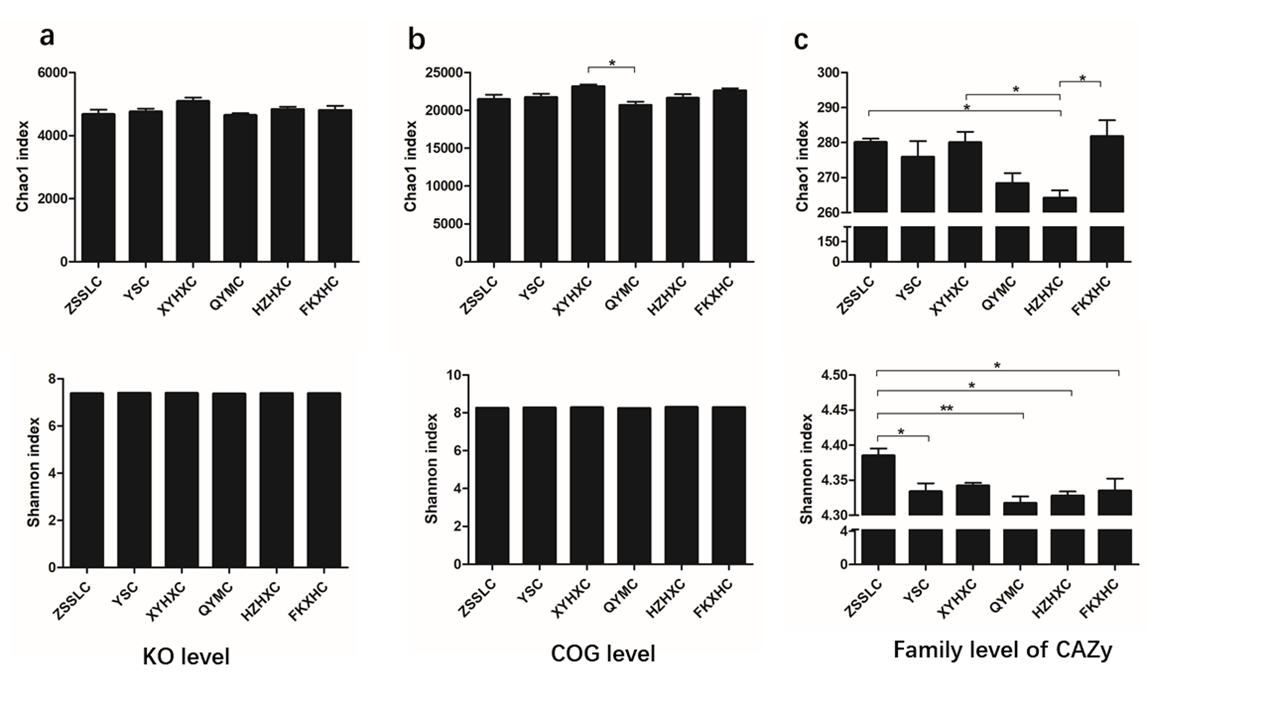


Figure S2. Alpha diversity (chao1 and shannon indies) analysis of **(a)** KO level, **(b)** COG level and **(c)** family level of CAZy. Data are expressed as means ± SEM, **p* < 0.05; ***p* < 0.01; ****p* < 0.001.
